# Do vigorous-intensity and moderate-intensity physical activities reduce mortality to the same extent? A systematic review and meta-analysis

**DOI:** 10.1136/bmjsem-2020-000775

**Published:** 2020-10-05

**Authors:** Juan Pablo Rey Lopez, Angelo Sabag, Maria Martinez Juan, Leandro F M Rezende, Maria Pastor-Valero

**Affiliations:** 1 Prevention Research Collaboration, Sydney School of Public Health, University of Sydney, Sydney, Australia; 2 i+HEALTH Research Group, Department of Health Sciences, Universidad Europea Miguel de Cervantes, Valladolid, Spain; 3 NICM Health Research Institute, Western Sydney University, Sydney, Australia; 4 Salud Pública, Historia de la Ciencia y Ginecología, Universidad Miguel Hernández, Elche, Spain; 5 Universidade Federal de Sao Paulo, Escola Paulista de Medicina, Departamento de Medicina Preventiva, Sao Paulo, Brazil; 6 Centro de Investigación Biomédica en Red en Epidemiología y Salud Pública (CIBERESP), Madrid, Spain

**Keywords:** Epidemiology, physical activity, prevention

## Abstract

**Objective:**

To examine whether vigorous-intensity physical activity confers additional reductions on all-cause and cause-specific mortality compared with moderate-intensity physical activity.

**Design:**

A systematic review (registered in PROSPERO CRD42019138995) and meta-analysis.

**Data sources:**

Three electronic databases up to April 14 2020.

**Eligibility criteria:**

Inclusion criteria were prospective studies that contained information about (1) moderate-intensity (3–5.9 metabolic equivalent tasks (METs)) and vigorous-intensity (≥6 METs) physical activities and (2) all-cause and/or cause-specific mortality. Exclusion criteria were prospective studies that (1) exclusively recruited diseased patients (eg, hypertensive patients and diabetics) or (2) did not account for total physical activity in their multivariable models (3) or did not adjust or exclude individuals with comorbidities at baseline or (4) used physically inactive participants as reference group.

**Results:**

Five studies (seven cohorts using sex-specific results) were pooled into a meta-analysis. For all-cause mortality and controlling by total physical activity, vigorous-intensity physical activity (vs moderate) was not associated with a larger reduction in mortality (HR 0.95, 95% CI 0.83 to 1.09). After the exclusion of one study judged with critical risk of bias (Risk Of Bias in Non randomized Studies, ROBINS tool) from meta-analysis, results remained similar (HR 0.98, 95% CI 0.85 to 1.12). Due to the limited number of studies, meta-analyses for cancer and cardiovascular mortality were not performed.

**Conclusions:**

Prospective studies suggest that, for the same total physical activity, both vigorous-intensity and moderate-intensity physical activities reduce all-cause mortality to the same extent. However, absence of evidence must not be interpreted as evidence of absence due to the existing methodological flaws in the literature.

## INTRODUCTION

Low physical activity in adult populations of industrialised nations is recognised as one of the main drivers of poor health.^1^ Globally, insufficient physical activity (aka physical inactivity) is considered the fourth leading cause of death,^2^ responsible for an estimated 5.3 million deaths per year.^3^ While many factors determine population health, it is well accepted that modern physical activity environments depart radically from preindustrialised settings. Motorised transportation, sedentary jobs, increased urbanisation and wide use of technology during leisure time divert from the physically active lifestyles, which were required for human survival in the past.^1^ However, there is a limited knowledge of the role of specific components of physical activity (ie, intensity) on different health outcomes across adulthood. The inconsistent findings obtained in prospective studies have led to conflicting views surrounding the importance of physical activity intensity for health.

On one hand, physical activity guidelines for health developed by expert groups in the USA,^4^ the UK,^5^ Australia^6^ or from the WHO^7^ recommend that adults should accumulate at least 150 min of moderate-intensity activity (3–5.9 metabolic equivalent tasks, METs) or 75 min of vigorous-intensity activity (≥6 METs) per week or a combination of both intensities, where 1 min of vigorous-intensity activity counts as 2 min of moderate-intensity activity. This implies that, when the overall activity energy expenditure is held constant, both moderate-intensity and vigorous-intensity activities may yield similar benefits for health. On the other hand, during the last decade, there has been a growing scientific interest among exercise physiologists into the potential of high-intensity vigorous training programmes as a time-efficient strategy to improve health.^8^ Compared with activities of lower intensity (eg, prolonged moderate-intensity activities), some scientists claim that vigorous-intensity activities (often of shorter duration) elicit comparable or superior health benefits to lower-intensity activities due to, among other reasons, larger improvements in cardiorespiratory fitness.^8–19^ Nonetheless, to date, few observational studies have studied the effect of vigorous-intensity activities on mortality compared with moderate intensity, after controlling for total physical activity.^20–25^ While some prospective studies reported larger risk reductions on all-cause^23–25^ and cardiovascular disease (CVD) mortality in participants with higher vigorous-intensity activity,^21^ another study observed inconsistent associations (of vigorous-intensity vs moderate-intensity activities) on CVD mortality between sexes and a lower all-cause mortality in men.^22^

The aim of this systematic review was to compare vigorous-intensity versus moderate-intensity physical activities in relation to all-cause and cause-specific mortality, after adjusting for total physical activity.

## METHODS

This systematic review was conducted and reported based on the Preferred Reporting Items for Systematic Reviews and Meta-analysis statement,^26^ and the review protocol was registered in PROSPERO (CRD42019138995).

### Data source and search

Two authors (AS and JPRL) in collaboration with a professional librarian developed the search strategy. A comprehensive electronic database search was conducted in Scopus, Web of Science and EMBASE via Ovid, for studies published from inception until April 14, 2020 (search strategy is shown in online supplemental appendix A). Duplicates of studies were removed using EndNote (Clarivate Analytics, Philadelphia, Pennsylvania, USA).

10.1136/bmjsem-2020-000775.supp1 Supplementary data

The search was restricted to adult human studies and publications in English, Spanish or Portuguese. In addition, reference lists of included studies, and systematic and narrative reviews about physical activity and longevity/mortality were screened for searching additional studies.

### Study selection

For the systematic review, the eligibility criteria were as follows: prospective cohort studies involving adults (≥18 years) and comparing vigorous-intensity (≥6 METs) to moderate-intensity (ie, activities that produced an energy expenditure of 3–5.9 METs) physical activities for all-cause and cause-specific (cardiovascular or cancer) mortality. Prospective studies were excluded when they were exclusive if conducted in diseased patients (eg, hypertensive patients and diabetics) or did not account for total physical activity in the multivariable models. Representative, population-based cohort studies (which have a small proportion of participants with comorbidities) were considered eligible only if their effect sizes were adjusted for comorbidities or diseased individuals at baseline (ie, CVD risk factors, heart disease and cancer).

To be included in the meta-analysis, we only selected those studies that compared mortality between participants reporting vigorous-intensity versus moderate-intensity physical activities (ie, studies including inactive participants as reference group were excluded).

### Screening and data extraction

Two reviewers (AS and MMJ) independently screened the titles and abstracts to remove irrelevant studies. During the selection stage, a pair of reviewers (AS and MMJ) independently read the full-text articles and made the selection based on the eligibility criteria. Discrepancies during the screening or selection stages were resolved by a third author (JPRL).

Information on the characteristics of the included studies were extracted independently by two reviewers (MMJ and JPRL): (1) authors and year of publication; (2) country, sample size, sex and age at baseline; (3) follow-up time; (4) measurement of physical activity; (5) outcome variables; (6) covariates included in the maximally adjusted model; (7) exposure reference group; (8) main results (eg, maximally adjusted HR and 95% CI).

### Risk of bias assessment

Risk of bias of included studies was appraised by two authors (LFMR and JPRL) using the ROBINS-I tool (‘Risk Of Bias In Non-randomised Studies of Interventions’).^27^ This approach for evaluating risk of bias in observational studies covers seven domains through which bias might be introduced into interventions (or exposure groups) of studies that did not use randomisation to allocate participants to comparison groups. The tool includes a list of signalling questions for the following domains: (1) bias due to confounding, (2) bias in selection of participants into the study, (3) bias in classification of interventions, (4) bias due to deviations from intended interventions, (5) bias due to missing data, (6) bias in measurement of outcomes and (7) bias in selection of the reported result. Domain level judgements about risk of bias are conceived hierarchically: (1) low risk of bias (where the study is comparable to a well-performed randomised trial with regard to that domain), (2) moderate risk of bias (where the study is sound for a non-randomised study but cannot be considered comparable to a well-performed randomised trial with regard to that domain), (3) serious risk of bias (the study has some important problems in that domain), (4) critical risk of bias (the study is too problematic in that domain to provide any useful evidence on the effects of the intervention) and (5) no information on which to base a judgement about risk of bias for that domain. Any disagreement was discussed between the two authors and, in case of further disagreement, a third author (MP-V) was recruited for a final judgement.

### Data analysis

Data from individual studies were pooled (HRs and 95% CI and two-sided pvalues) using a random effects model (Mantel-Haenszel weights by the *metan* package) to estimate the overall summary HR for all-cause and cause-specific mortality comparing vigorous-intensity versus moderate-intensity physical activity. In addition, to assess the impact of heterogeneity on the summary effect (under the random effect model), prediction intervals were calculated. When results of the original studies reported several multivariable models, only the data from the maximally adjustment model was considered. When authors reported several categories of vigorous intensity in their statistical analyses, only the group with the highest proportion of vigorous-intensity physical activity to total physical activity was extracted. Studies that reported results for men and women were included in the analysis as two separate cohorts. Heterogeneity was quantified using the I^2^ statistic and the χ² Cochran Q-test.^28^ The CI for the I^2^ measure was derived using the Q statistic and the corresponding degrees of freedom. Publication bias was examined using the asymmetry tests (graphically using the *metafunnel* package) and by the contour-enhanced funnel plots (using the *confunnel* package).^29^ Sensitivity analyses to determine whether a particular study largely accounted for heterogeneity were explored by removing one by one study from the meta-analysis. All analyses were performed using Stata, version 15 (College Station, Texas, USA).

## RESULTS

### Study selection

**Figure 1:**
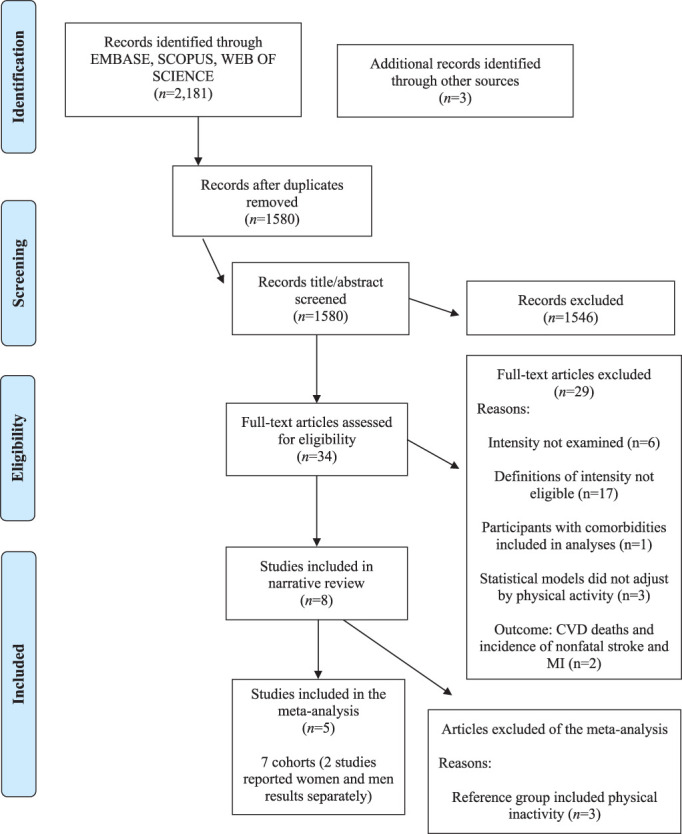
Preferred Reporting Items for Systematic Reviews and Meta-analysis flow diagram for search strategy. Prospective studies.

The search strategy identified 2181 records, with 1580 articles remaining after duplicates were removed. Of these, 1546 were excluded after screening titles and abstracts because they did not meet predetermined selection criteria (ie, conference papers and cross-sectional studies). Of the 34 full-text articles retrieved, 29 studies were excluded from the systematic review (figure 1 and online supplemental appendix B) because physical activity intensity was not examined (n=6), the definitions used for moderate-intensity or vigorous-intensity differed of the standard definitions of the literature (n=17), comorbidities were not controlled in the reported analyses (n=1), total physical activity was not included as a covariate into the model (n=3) and outcome included both fatal and non-fatal CVD (n=2). Finally, three articles included in the systematic review were not summarised in the meta-analysis^30–32^ because physically inactive participants were assigned as reference group.

10.1136/bmjsem-2020-000775.supp2 Supplementary data

### Study characteristics

Participant characteristics from eight studies meeting the eligibility criteria of the systematic review are summarised in table 1. A total of five studies were included in the meta-analysis (seven cohorts using sex-specific results).^22–25 33^ All studies were carried out in high-income countries: Germany,^31^ Finland,^33^ the UK,^25^ Australia,^23^ Japan^24^ and the USA.^22 30 32^

**Table 1:** Main descriptive characteristics of the prospective studies (n=8) identified in the systematic review

	Population			
Author/year/ref.	Country	Sample size (n)	Sex	Age at baseline (years)	Follow-up (years unless otherwise stated)	Measurement of the exposure	Type of the outcome	Covariates (maximally adjusted model)	Reference group	Main results (HR, 95% CI)
Leitzmann *et al* 2007^30^	USA	252 925	Both	Range: 50–71	1 265 347 person-years	Questionnaire. One questionnaire at baseline (vigorous-intensity activity) and a second one 6 months later. *Moderate intensity:* average time spent each week in activities of at least moderate intensity during the previous year. *Vigorous intensity:* frequency of vigorous intensity per week	All-cause mortality CVD mortality Cancer mortality	Age, sex, BMI, smoking, race/ethnicity, education, marital status, family history of cancer, menopausal hormone therapy, aspirin use, multivitamin use, intakes of vegetables, fruit, red meat, and alcohol; and moderate-intensity or vigorous-intensity exercise	No physical activity	All-cause mortality: Leisure time Moderate (>7 h/week): 0.68 (0.63 to 0.74) Vigorous (≥5 times/week): 0.71 (0.66 to 0.77) CVD mortality: Leisuretime Moderate: 0.65 (0.57 to 0.75) Vigorous: 0.71 (0.62 to 0.82) Cancer mortality: Leisure time Moderate: 0.83 (0.74 to 0.93) Vigorous: 0.95 (0.85 to 1.07)
Autenrieth *et al* 2011^31^	Germany	4672	Both	Mean (SD): 49.6 (14.0)	17.8 (median)	Questionnaire. Once at baseline. Time usually spent per week on PA during *work, transportation, household and leisure time* over the past year. No adjustments for other types of physical activity in total activity	All-cause mortality CVD mortality Cancer mortality	Sex, BMI, systolic blood pressure, total-to-HDL cholesterol ratio, education, smoking status, alcohol consumption, myocardial infarction, stroke, diabetes, cancer, self-reported limited physical activity due to health problems and other domains of physical activity	No physical activity	All-cause mortality: Transportation Moderate: 1.16 (1.00 to 1.35) Vigorous: 0.95 (0.80 to 1.14) Leisure time PA Moderate: 0.78 (0.60 to 0.95) Vigorous: 0.48 (0.36 to 0.65) Total PA Moderate: 0.91 (0.76 to 1.10) Vigorous: 0.73 (0.59 to 0.90) CVD mortality: Transportation Moderate: 1.23 (0.98 to 1.55) Vigorous: 1.02 (0.78 to 1.34) Leisuretime PA Moderate: 0.97 (0.71 to 1.33) Vigorous: 0.50 (0.31 to 0.79) Total PA Moderate: 0.98 (0.74 to 1.30) Vigorous: 0.75 (0.55 to 1.03) Cancer mortality: Transportation Moderate: 1.19 (0.91 to 1.56) Vigorous: 0.89 (0.64 to 1.24) Leisure time PA Moderate: 0.56 (0.40 to 0.77) Vigorous: 0.36 (0.23 to 0.59) Total PA Moderate: 0.81 (0.60 to 1.11) Vigorous: 0.62 (0.43 to 0.88)
Zhao *et al* 2020^32^	USA	88 140	Both	Range: 40–85	9 (median)	Questionnaire. Once at baseline. Frequency and duration of leisure time PA. Vigorous (ex, running, faster cycling, competitive sports); light and moderate (brisk walking, dancing, gardening)	All-cause mortality CVD mortality Cancer mortality	Age, sex, race/ethnicity, moderate or vigorous PA, education, marital status, BMI, smoking, alcohol intake	No physical activity reported in 6451 participants of the reference group (n=57 889 were included in the group: 0 min per week of vigorous)	All-cause mortality: Leisure time Vigorous (≥600 min/week): 0.58 (0.46 to 0.73) CVD mortality: Leisuretime Vigorous (≥600 min/week): 0.66 (0.40 to 1.07) Cancer mortality: Leisure time Vigorous (≥600 min/week): 0.61 (0.42 to 0.89)
Lahti *et al* 2014^33^	Finland	6429	Both	Range: 40–60	12 (mean)	Questionnaire. Once at baseline. Time usually spent per week on PA during *leisure time including transportation* within the previous 12 months	All-cause mortality	Age, gender, occupational social class, physical and mental strenuousness of work, smoking and drinking problems, body mass index, physical functioning, mental health, limiting long-standing illness and volume of physical activity	Low moderate	All-cause mortality: Leisure time PA (including transportation) High moderate: 0.73 (0.49 to 1.1) Vigorous: 0.55 (0.32 to 0.94)
Shiroma *et al* 2014^22^	USA	7979 (M) 38 671 (F)	Both	M: mean (SD) 66.1 (7.7) F: mean (SD) 54.6 (7.0)	M: 17.3 (mean) F: 16.4 (mean)	Questionnaire. Number of self-reported the number of flights of stairs climbed daily and the number of blocks walked daily and frequency and duration spent doing sports or recreational activities. Physical activity questionnaires 3 times in men (baseline, 5 years later, 10 years later); in womens at baseline and every 2–3 years	All-cause mortality CVD mortality	M: Age, MVPA, smoking status, dietary factors, alcohol consumption, BMI, high cholesterol and hypertension F: Age, MVPA, BMI, high cholesterol, hypertension, clinical trial randomisation, smoking status, dietary factors, alcohol consumption, postmenopausal status, hormone therapy, and parental history of myocardial infarction	≤10% of MVPA performed at vigorous intensity	All-cause mortality: Leisure time (>75% MVPA) Vigorous (M) 0.95 (0.82 to 1.09) Vigorous (F): 1.23 (1.11 to 1.36) CVD mortality: Leisure time (>75% MVPA) Vigorous (M): 0.83 (0.63 to 1.10) Vigorous (F): 1.15 (0.90 to 1.45)
Gebel *et al* 2015^23^	Australia	204 542	Both	Range: 45–75	6.52 (mean)	Questionnaire. Once at baseline. Participants reported sessions (bouts of at least 10 min) and duration of walking and moderate-intensity and vigorous-intensity activities in the past week	All-cause mortality	Age, sex, education, marital status, urban/rural residence, smoking status, weight status, physical function, alcohol consumption, fruit and vegetable intake, and total weighted volume of MVPA	0% of MVPA performed at vigorous intensity	All-cause mortality: Leisure time (≥30% MVPA) Vigorous (all sample) 0.87 (0.81 to 0.93) Vigorous (M) 0.86 (0.79 to 0.94) Vigorous (F) 0.89 (0.80 to 0.99) No cardiometabolic diseases (≥30% MVPA) Vigorous (all) 0.86 (0.79 to 0.94)
Kikuchi *et al* 2018^24^	Japan	83 454	Both	M: mean (SD) 61.5 (7.4) F: mean (SD) 62.0 (7.6)	10.8 (mean)	Questionnaire. Three questionnaires: baseline, after 5 years and after 10 years. Frequency, intensity and duration of physical activity during leisure time	All-cause mortality	Age, sex, public health centres, smoking, drinking, BMI, diabetes history, hypertension status and MVPA	Physically inactive (<450 MET-min/week) 0% of MVPA performed at vigorous intensity	All-cause mortality: Leisure time Vigorous (M) 0.74 (0.62 to 0.89) Vigorous (F) 0.74 (0.58 to 0.94) All-cause mortality: Leisure time (≥30% MVPA) Vigorous (M) 1.01 (0.84 to 1.22) Vigorous (F) 1.04 (0.81 to 1.33)
Rey-Lopez *et al* 2019^25^	UK	64 913	Both	M: mean (SD) 49.8 (13.6)	9.0 (mean)	Questionnaire. Once at baseline. Frequency, intensity and duration of physical activity during leisure time (sports and exercise); duration, frequency and pace of walking; and domestic physical activity	All-cause mortality CVD mortality Cancer mortality	Age, sex, education, smoking, alcohol consumption, total weighted volume of MVPA, long-standing illness, BMI and psychological distress	0% of MVPA performed at vigorous intensity	Excluding the first 24 months of follow-up: All-cause mortality: Leisure time plus domesticPA and walking (≥30% MVPA) Vigorous (all) 0.85 (0.75 to 0.95) CVD mortality: Leisure time plus domestic PA and walking (≥30% MVPA) Vigorous (all) 0.88 (0.69 to 1.11) Cancer mortality: Leisure time plusdomestic PA and walking (≥30% MVPA) Vigorous (all) 0.88 (0.73 to 1.07)

BMI, body mass index; MVPA, moderate-intensity to vigorous-intensity physical activity; PA, physical activity.

We contacted by email with Kikuchi *et al* ^24^ as they reported a mistake in their publication. In the upper CI of men for (≥30% MVPA), the correct upper CI value is 1.22, instead of the reported value of 0.80.

The sample size of the studies ranged between 4672 and 204 542 participants. All studies included both men and women. Age of participants at the baseline ranged between 40 and 85 years. Average follow-up time varied from 6.52 years to 17.8 years. All studies measured participants’ physical activity through questionnaires. A total of five studies measured habitual physical activity (over the span of a year, whereas three studies collected physical activity data during the last week before the survey). Although one study provided detailed information on physical activity in several domains,^30^ most studies reported information on leisure time physical activity only. Reference groups of exposure were defined as follows: a physically inactive group (two studies),^30 31^ a combination of both physically inactive and active group,^32^ a low moderate-intensity group,^33^ 0% vigorous-intensity physical activity to total MVPA performed at vigorous intensity (three studies)^23–25^ and ≤10% of vigorous intensity to MVPA performed at vigorous intensity (one study).^22^ Regarding the outcome, all studies included all-cause mortality, five CVD mortality^22 25 30–32^ and four cancer mortality.^25 30–32^

### Risk of bias of prospective studies included in the meta-analysis

**Figure 2:**
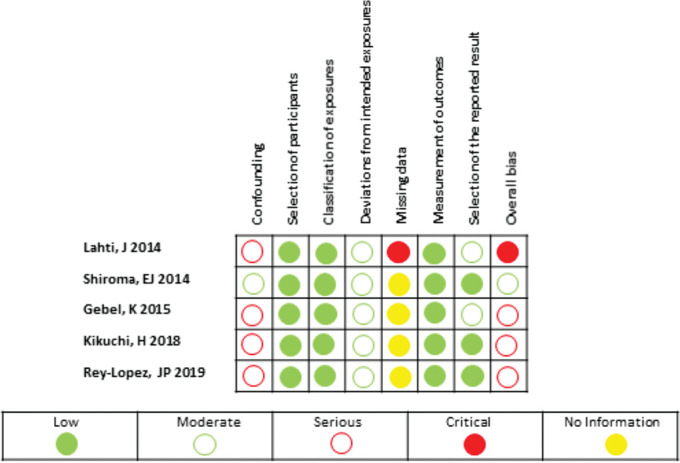
Risk of bias judgement using ROBINS-I.

In stage one, ROBINS-I requires the reviewers to specify the review question in order to emulate a hypothetical pragmatical randomised trial. Accordingly, healthy adults were defined as participants. The experimental intervention was vigorous-intensity physical activity and the comparator was moderate-intensity physical activity. Outcomes included all-cause mortality, cardiovascular mortality or cancer mortality. Confounders were age, sex, smoking, adiposity, alcohol consumption, dietary factors and individual-level socioeconomic factors. Online supplemental appendix C shows the results (consensus responses) obtained by the reviewers with the risk of bias judgements using ROBINS-I. For the confounding domain, four studies were judged as serious risk of bias and one with moderate risk of bias (figure 2). For three domains (selection of participants, classification of the exposures and measurement of the outcomes), all studies were judged as low risk of bias. For the domain deviations from intended exposures, all studies were evaluated as moderate risk of bias. Regarding the missing data domain, four studies were judged as no information while one study was scored as critical risk of bias. For the selection of the reported results domain, two studies were scored as low risk of bias and three as moderate risk. Finally, for the assessment of overall bias of each study, three studies were judged as serious risk of bias, one was judged as critical risk of bias and one as moderate risk of bias.

10.1136/bmjsem-2020-000775.supp3 Supplementary data

### Meta-analysis of vigorous-intensity versus moderate-intensity physical activity and all-cause mortality

**Figure 3:**
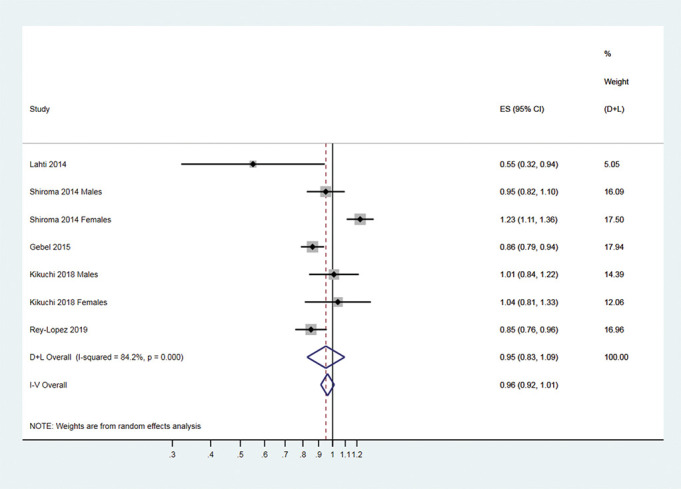
Forest plot summary of association between vigorous-intensity and moderate-intensity physical activities and all-cause mortality.

Five prospective studies^22–25 33^ (seven cohorts after using the sex-specific data) evaluated the association between vigorous-intensity physical activity (vs moderate-intensity) and all-cause mortality. Compared to moderate-intensity physical activity, vigorous-intensity physical activity was not associated with all-cause mortality (HR 0.95, 95% CI 0.83 to 1.09; I^2^ 84.2%, 95% CI 69%-92%; prediction interval 0.60–1.51) (figure 3). The exclusion of one study judged with a critical risk of bias from the meta-analysis^33^ did not change the direction of the association nor the heterogeneity (HR 0.98, 95% CI 0.85 to 1.12; I^2^ 85.2%, 95% CI 70% to 93%; prediction interval 0.61–1.51) (online supplemental appendix D). The exclusion of a large study with high risk of reverse causation from the meta-analysis did not change the results (HR 0.97, 95% CI 0.82 to 1.14; I^2^ 82.3%, 95% CI 62% to 92%); prediction interval 0.57–1.66) (online supplemental appendix E).

10.1136/bmjsem-2020-000775.supp5 Supplementary data

10.1136/bmjsem-2020-000775.supp4 Supplementary data

### Vigorous-intensity versus moderate-intensity physical activities and CVD mortality

Two studies^22 25^ (three cohorts after using the results available by sex) evaluated the association between vigorous-intensity physical activity and CVD mortality. Compared to moderate-intensity physical activity, women who reported the highest proportion of vigorous to total physical activity had 15% higher risk of CVD mortality (HR 1.15, 95% CI 0.90 to 1.45).^22^ In men, the highest proportion of vigorous-intensity physical activity to total physical activity was associated with lower CVD mortality (HR 0.83, 95% CI 0.63 to 1.10).^22^ Similarly, in 64 913 men and women from England and Scotland, the HR for CVD mortality in participants engaged in the highest proportion of vigorous-intensity physical activity was 0.88 (95% CI 0.69 to 1.11).^25^

Zhao *et al* ^32^ found a HR for CVD mortality of 0.66 (95% 0.40 to 1.07) when comparing participants with the highest proportion of vigorous-intensity activity to total physical activity vs 0% vigorous-intensity activity group (yet the reference group included both physically inactive and active participants).

### Vigorous-intensity vs moderate-intensity physical activity and cancer mortality

One study examined the association between vigorous-intensity physical activity (vs moderate) and cancer mortality.^25^ Compared to moderate-intensity (but not vigorous-intensity) physical activity, the HR for cancer mortality in the group engaged in the highest proportion of vigorous-intensity physical activity was 0.88 (95% CI 0.73 to 1.07).

Two additional prospective studies compared the association of vigorous-intensity physical activity (vs physical inactivity) and cancer mortality. Leitzmann *et al* (2007),^30^ in a sample of 252 925 middle-aged participants, found a HR of 0.95 (95% CI 0.85 to 1.07) when comparing vigorous group with moderate group. Another study conducted in 4672 middle-age adults during 17.8 years of follow-up showed a lower risk of cancer mortality in participants reporting vigorous-intensity versus moderate-intensity physical activities during leisure time (HR 0.36 95% CI 0.23 to 0.59).^31^ Finally, in a recent study from Zhao *et al*,^32^ a lower cancer mortality rate was observed in participants with the highest amount of vigorous-intensity activity (HR 0.61 95% 0.42 to 0.89) versus 0% vigorous-intensity activity. Nonetheless, the reference group used included both physically inactive and active participants.

### Publication bias

**Figure 4:**
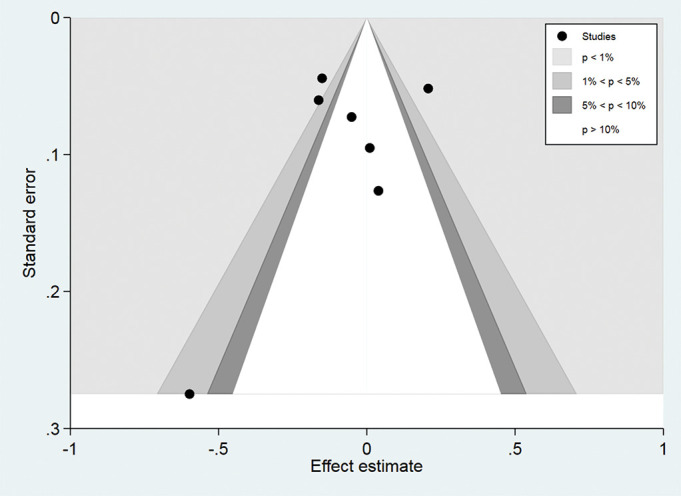
Contour-enhanced funnel plot in all cohort studies.

**Figure 5:**
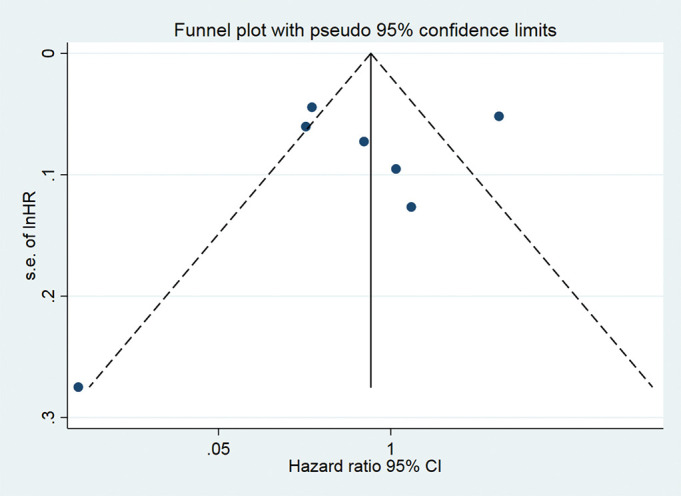
Funnel plot with pseudo 95% CI for identifying publication bias in all cohort studies.

As shown in figure 4, we found asymmetry in the funnel plot (as one study was located at the bottom of the funnel plot). However, after the inspection of the contour-enhanced funnel plots, we found that the asymmetry in the funnel plot was caused not only for publication bias, because studies were not missing in areas of high statistical significance (figure 5).

## DISCUSSION

Results from five prospective studies (seven different cohorts) suggest that, for the same volume of physical activity, vigorous-intensity and moderate-intensity physical activities reduce mortality to the same extent. There was a 5% lower all-cause mortality among adults reporting regular vigorous-intensity physical activity compared with those reporting moderate-intensity physical activity. When we excluded one study judged as critical risk of bias (overall),^33^ vigorous-intensity activity was associated with a 2% lower all-cause mortality (online supplemental appendix D). On the other hand, evidence was unclear for CVD mortality cancer mortality due to the lower number of studies.

Our findings are in agreement with the current physical recommendations for health in adults developed in countries such as the USA, Australia and the UK as well as the WHO. Remarkably, all the above-mentioned recommendations do not prioritise vigorous over moderate intensity to maximise the health benefits. Nonetheless, during the last decade programmes of short duration, vigorous-intensity activities have received considerable scientific attention among clinical researchers interested in the cardiometabolic benefits of exercise.^8^ Given that high-intensity training studies have been conducted in laboratory settings during a short period of time, it is unknown whether vigorous-intensity activities maintained throughout life may offer larger health benefits compared with energy-matched physical activity of lower intensity. We found five prospective studies that aimed to close this gap of knowledge; yet, the existence of biases inherent to epidemiological studies challenges their interpretation. As depicted in figure 2, four of five studies included in this review were deemed as having a serious risk of bias for the confounding domain. To evaluate the risk bias in the confounding domain and following ROBINS-I recommendations, we specified a list of covariates (by expert knowledge) that requires controlling of confounding factors such as age, sex, smoking, adiposity, alcohol consumption, dietary factors and individual-level socioeconomic factors. However, we found that most studies did not control for the whole list of mentioned covariates in their multivariable models.

A well-known limitation of epidemiological studies involving physical activity and mortality is reverse causation. To minimise this problem, we pooled prospective studies that accounted for the diagnosis of any disease in the multivariable model and/or excluded those with diseased participants. Nevertheless, some studies in the literature failed to account for reverse causation. For example, in the main results reported by Gebel *et al*,^23^ participants diagnosed with cardiometabolic diseases at baseline were included in the analysis. Therefore, we only included in the meta-analysis estimates obtained from participants without cardiometabolic diseases at baseline.^23^ Regarding other domains included in ROBINS-I, it is remarkable that most studies did not provide information about missing data. Missing data may occur among other reasons through loss to follow-up, incomplete data collection and exclusion from analysis by investigators. Bias (ie, the effect estimate obtained in the study is different from the one obtained if authors had a complete dataset) may arise if the reason for and/or the proportion of missing data differs according to groups being compared.

Although we observed some asymmetry in the funnel plot, the results obtained in the contour-enhanced funnel plot suggest that causes of the observed asymmetry were likely due to other factors rather than publication bias (ie, systematic differences between studies in the results of large and small prospective studies or differential methodological quality of the prospective studies identified).^29^ Furthermore, problems with the design of some prospective studies identified in the systematic review forced us to exclude them from the meta-analysis. For example, three studies (included in the narrative review) evaluated moderate-intensity and vigorous-intensity physical activities but employed as counterfactual reference groups: people with no physical activity^30 31^ or participants with no physical activity and some amount of non-vigorous-intensity physical activity.^32^ These analytical decisions may overestimate the benefits of vigorous-intensity physical activity on mortality. Evidence of the latter may be found in the analyses of Kikuchi *et al*,^24^ where a significant protective effect was reported using the physically inactive group (450 MET min/week) while no association was found using 0% of vigorous intensity to MVPA.

From an evolutionary perspective, it has been suggested that endurance running was instrumental in the survival of *Homo sapiens.* ^34^ This opinion was, however, drawn on a narrative review of physiological and anatomical bases of endurance running capabilities of humans versus other primates. An observational study in modern hunter-gatherers (ie, Hadza) showed that adults tend to accumulate (per day) over 135 min of moderate-intensity to vigorous-intensity physical activities (measured by accelerometry), mostly at moderate intensity.^1^ In a recent study that compared cardiovascular adaptations induced by different types of exercise among humans, gorillas and chimpanzees, authors claimed that humans have evolved multisystem capabilities mainly matched for regular moderate-intensity endurance physical activity.^35^ Low volume strength and power physical activities (which can be categorised as vigorous-intensity activities) were an occasional form of physical activity in our ancestors. Interestingly, cardiological adaptations induced by strength and power exercise seem less cardioprotective (at least structurally) compared with programmes of moderate-intensity physical activity.^35^

Although our data indicates that both vigorous-intensity and moderate-intensity physical activities in adulthood may reduce mortality to the same extent, this finding should not downplay the key role of physical activity to improve individual and population health. Some estimates suggest that, worldwide, physical inactivity causes 6% of coronary heart disease, 7% of type 2 diabetes, 10% of breast cancer and 10% of colon cancer.^3^ The finding that either moderate-intensity activity or vigorous-intensity activity may provide similar reductions on mortality (for the same physical activity energy expenditure) is, if confirmed, good news because physical inactive population groups can find it difficult to attain and maintain intensities of vigorous intensity.

To our knowledge, this is the first systematic review and meta-analysis designed to evaluate whether vigorous-intensity activities (vs moderate-intensity) may provide additional reductions on mortality after controlling for total physical activity. As the results of meta-analysis are only as valid as the quality of the studies included,^36^ we performed risk of bias assessments of the selected literature using ROBINS-I.

### Limitations

We acknowledge several limitations. First, the exposure variable (physical activity) was assessed by questionnaires, which are less accurate than objective methods of physical activity measurement.^37^ Future epidemiological studies will undoubtedly benefit new technological developments to evaluate objectively physical activity level in large populations. In this sense, a recent prospective study conducted in 16 741 women evaluated the association of number and intensity of steps (by accelerometry) and mortality.^38^ Authors concluded that stepping higher in the intensity level was not related to lower mortality rates after accounting for total steps for a day.^38^ Second, only one measurement of physical activity was collected in all epidemiological studies identified (at baseline), which may have led to regression dilution bias. Physical activity levels tend to decrease with age, which in theory could attenuate a protective effect on mortality of vigorous intensity due to transitions toward inactive lifestyles. Further information on physical activity changes during adulthood will increase the quality of the available epidemiological evidence. Third, a low number of studies met our strict eligibility criteria. A reason for additional concern is the lack of epidemiological data about CVD or cancer mortality. Our review, therefore, highlights the urgent need to evaluate cause-specific mortality in future well-designed epidemiological studies. Finally, a small number of cohorts were identified and pooled into the meta-analysis and subgroup or meta-regression analyses were discarded (due to low statistical power) to explore the sources of heterogeneity.

## CONCLUSION

The epidemiological evidence available indicates that vigorous-intensity and moderate-intensity physical activity may reduce mortality to the same extent, after controlling by the volume of physical activity. However, the absence of the evidence must not be interpreted as evidence of absence. This systematic review, indeed, shows that there are still very few prospective studies specifically designed to examine the role of intensity of physical activity on mortality, and important methodological flaws were observed in the literature (ie, risk of bias due to confounding and lack of objective assessments of physical activity).

**Summary box**

**What is already known in this subject?**

- Low physical activity level in adult populations is recognised as one of the main drivers of poor population health. However, it is currently unclear whether vigorous-intensity physical activity may lead to larger reductions in mortality compared with moderate-intensity physical activity.

**What are the new findings?**

- The best synthesis of the epidemiological evidence shows similar risk reductions on all-cause mortality in participants that reported the highest proportion of vigorous-intensity activity versus moderate-intensity activity.
- A lower number of prospective studies have evaluated the impact of physical activity intensity on cause-specific mortality (CVD and cancer), and their findings do not convincingly support the hypothesis that vigorous intensity may produce larger benefits on mortality over moderate intensity.
- Future epidemiological studies on intensity of physical activity and mortality will require better study designs. For example, the use of objective tools to assess physical activity includes dietary variables as covariates, evaluates cause-specific mortality and informs about the proportion of missing data from participants.
