# Supplementary material for: Do vigorous-intensity and moderate-intensity physical activities reduce mortality to the same extent? A systematic review and meta-analysis
Source: BMJ Open Sport Exerc Med. 2020 Oct 5;6(1):e000775. doi: 10.1136/bmjsem-2020-000775 (PMC7610342; doi:10.1136/bmjsem-2020-000775)
Supplement: Supplementary data [file bmjsem-2020-000775s001.pdf]

Appendix A: Search strategy of the systematic review

We searched for articles indexed in *Scopus, Web of Science and EMBASE via Ovid* that were published from inception until 14th April 2020. The search was restricted to adult human studies and publications in English, Spanish or Portuguese language. In addition, reference lists of studies included in systematic review and reviews about physical activity and longevity or mortality were screened for additional studies.

Keywords used in the search strategy: (physical activity, exercise, physical exercise, physical fitness, walk*, jog*, run*) and (moderate physical activity/2, moderate vigorous/3, vigorous phys* acti*, strenuous, high intensity/3, metabolic equivalent) and (mortality, all-cause mortality/3, cardiovascular mortality, cancer mortality) and (longitudinal, cohort, prospective).
