# Supplementary material for: Do vigorous-intensity and moderate-intensity physical activities reduce mortality to the same extent? A systematic review and meta-analysis
Source: BMJ Open Sport Exerc Med. 2020 Oct 5;6(1):e000775. doi: 10.1136/bmjsem-2020-000775 (PMC7610342; doi:10.1136/bmjsem-2020-000775)
Supplement: Supplementary data [file bmjsem-2020-000775supp002.pdf]

Appendix B: Full-text articles excluded of the systematic review (eligibility stage) and reasons

First author	Title	Reasons
Tarp J et al. 2020	Accelerometer-measured physical activity and sedentary time in a cohort of US adults followed for up to 13 years: The influence of removing early follow-up on associations with mortality.	Definition of intensity did not meet eligibility criteria
Liu Q et al. 2020	Beneficial effects of moderate to vigorous physical activity on cardiovascular disease among Chinese adults.	Outcome included CVD deaths and incidence of nonfatal stroke or myocardial infarction
Cheung et al. 2017	Leisure-Time Physical Activity and Cardiovascular Mortality in an Elderly Population in Northern Manhattan: A Prospective Cohort Study	Definition of intensity did not meet eligibility criteria
Almeida et al. 2014	150 minutes of vigorous physical activity per week predicts survival and successful ageing: a population-based 11-year longitudinal study of 12 201 older Australian men	Definition of intensity did not meet eligibility criteria
Arem et al. 2015	Leisure Time Physical Activity and Mortality A Detailed Pooled Analysis of the Dose-Response Relationship	Authors included in their analyses participants with comorbidities at baseline
Barengo et al. 2004	Low physical activity as a predictor for total and cardiovascular disease mortality in middle-aged men and women in Finland	Intensity of physical activity was not examined
Bayan-Bravo et al. 2019	The association of major patterns of physical activity, sedentary behavior and sleeping with mortality in older adults	Intensity of physical activity was not examined
Andersen et al. 2000	All-cause mortality associated with physical activity during leisure time, work, sports, and cycling to work	Intensity of physical activity was not examined
Borgundvaag et al. 2017	Objectively Measured Physical Activity and Mortality Risk Among American Adults	Definition of intensity did not meet eligibility criteria
Crespo et al. 2008	Physical activity and prostate cancer mortality in Puerto Rican men	Definition of intensity did not meet eligibility criteria
Dankel et al. 2016	Determining the Importance of Meeting Muscle-Strengthening Activity Guidelines: Is the Behavior or the Outcome of the Behavior (Strength) a More Important Determinant of All-Cause Mortality?	Intensity of physical activity was not examined
Dohrn et al. 2018	Accelerometer-measured sedentary time and physical activity-A 15 year follow-up of mortality in a Swedish population-based cohort	Definition of intensity did not meet eligibility criteria
Ensrud et al. 2014	Objective measures of activity level and mortality in older men	Definition of intensity did not meet eligibility criteria

Evenson et al. 2017	Accelerometry-Assessed Latent Class Patterns of Physical Activity and Sedentary Behavior With Mortality	Definition of intensity did not meet eligibility criteria
Haapanen et al. 1996	Characteristics of leisure time physical activity associated with decreased risk of premature all-cause and cardiovascular disease mortality in middle-aged men	Physical activity was not included as a covariate in the analyses
Hidalgo-Santamaria et al. 2018	Physical Activity Intensity and Cardiovascular Disease Prevention—From the Seguimiento Universidad de Navarra Study	Outcome included CVD deaths and incidence of nonfatal stroke or myocardial infarction
Holme et al. 2015	Increases in physical activity is as important as smoking cessation for reduction in total mortality in elderly men: 12 years of follow-up of the Oslo II study	Physical activity was not included as a covariate in the analyses
Hsu et al. 2018	Total Physical Activity, Exercise Intensity, and Walking Speed as Predictors of All-Cause and Cause-Specific Mortality Over 7 Years in Older Men: The Concord Health and Aging in Men Project	Definition of intensity did not meet eligibility criteria
Kopperstad et al. 2017	Physical activity is independently associated with reduced mortality: 15-years follow-up of the Hordaland Health Study (HUSK)	Definition of intensity did not meet eligibility criteria
Kujala et al. 1998	Relationship of leisure-time physical activity and mortality - The Finnish Twin Cohort	Definition of intensity did not meet eligibility criteria
LaMonte et al. 2018	Accelerometer-Measured Physical Activity and Mortality in Women Aged 63 to 99	Definition of intensity did not meet eligibility criteria
Laukkanen et al. 2011	Intensity of leisure-time physical activity and cancer mortality in men	Definition of intensity did not meet eligibility criteria
Lear et al. 2017	The effect of physical activity on mortality and cardiovascular disease in 130 000 people from 17 high-income, middle-income, and low-income countries: the PURE study	Definition of intensity did not meet eligibility criteria
Lee et al. 2014	Leisure-time running reduces all-cause and cardiovascular mortality risk	Definition of intensity did not meet eligibility criteria
Lee et al. 1995	Exercise intensity and longevity in men - the Harvard alumni health study	Definition of intensity did not meet eligibility criteria
Lee et al. 2016	Examining Non-Linear Associations between Accelerometer-Measured Physical Activity, Sedentary Behavior, and All-Cause Mortality Using Segmented Cox Regression	Physical activity was not included as a covariate in the analyses
Matthews et al. 2014	Physical Activity, Sedentary Behavior, and Cause-Specific Mortality in Black and White Adults in the Southern Community Cohort Study	Intensity of physical activity was not examined
Mok et al. 2016	Physical Activity Level and Colorectal Cancer Mortality	Intensity of physical activity was not examined

Williams et al. 2013	The Relationship of Walking Intensity to Total and Cause-Specific Mortality. Results from the National Walkers' Health Study	Definition of intensity did not meet eligibility criteria
