# Supplementary material for: Do vigorous-intensity and moderate-intensity physical activities reduce mortality to the same extent? A systematic review and meta-analysis
Source: BMJ Open Sport Exerc Med. 2020 Oct 5;6(1):e000775. doi: 10.1136/bmjsem-2020-000775 (PMC7610342; doi:10.1136/bmjsem-2020-000775)
Supplement: Supplementary data [file bmjsem-2020-000775supp003.pdf]

Appendix C: Risk of bias assessment- ROBINS-I tool

(the column “Description” indicates the consensus responses assigned by reviewers)

Lathi et al. 2014

Responses underlined in green are potential markers for low risk of bias, and responses in red are potential markers for a risk of bias. Where questions relate only to sign posts to other questions, no formatting is used.

Signalling questions	Description	Response options<br>Y: Yes; PY: Probably yes; PN: Probably no; N: No; NI: No information
**Bias due to confounding**		
1.1 Is there potential for confounding of the effect of intervention in this study?<br>**If N/PN to 1.1:** the study can be considered to be at low risk of bias due to confounding and no further signalling questions need be considered	Y. Diet was not included as a covariate.	Y / PY / PN / N
**If Y/PY to 1.1**: determine whether there is a need to assess time-varying confounding:		
1.2. Was the analysis based on splitting participants' follow up time according to intervention received?<br>**If N/PN**, answer questions relating to baseline confounding (1.4 to 1.6)<br>**If Y/PY**, go to question 1.3.	N	NA / Y / PY / PN / N / NI
1.3. Were intervention discontinuations or switches likely to be related to factors that are prognostic for the outcome?<br>**If N/PN**, answer questions relating to baseline confounding (1.4 to 1.6)<br>**If Y/PY**, answer questions relating to both baseline and time-varying confounding (1.7 and 1.8)	NA	NA / Y / PY / PN / N / NI

**Questions relating to baseline confounding only**		
1.4. Did the authors use an appropriate analysis method that controlled for all the important confounding domains?	N. Insufficient adjustment in the main reported analyses. Dietary factors were not included as confounders.	NA / Y / PY / PN / N / NI
1.5. **If Y/PY to 1.4**: Were confounding domains that were controlled for measured validly and reliably by the variables available in this study?	NA	NA / Y / PY / PN / N / NI
1.6. Did the authors control for any post-intervention variables that could have been affected by the intervention?	N	NA / Y / PY / PN / N / NI
**Questions relating to baseline and time-varying confounding**		
1.7. Did the authors use an appropriate analysis method that controlled for all the important confounding domains and for time-varying confounding?	NA	NA / Y / PY / PN / N / NI
1.8. **If Y/PY to 1.7**: Were confounding domains that were controlled for measured validly and reliably by the variables available in this study?	NA	NA / Y / PY / PN / N / NI
**Risk of bias judgement**	Serious. At least on important domain was not measured/controlled	Low / Moderate / Serious / Critical / NI
Optional: What is the predicted direction of bias due to confounding?	Favours experimental	Favours experimental / Favours comparator / Unpredictable

Bias in selection of participants into the study		
2.1. Was selection of participants into the study (or into the analysis) based on participant characteristics observed after the start of intervention?<br>**If N/PN to 2.1:** go to 2.4	N	Y / PY / PN / N / NI
2.2. **If Y/PY to 2.1**: Were the post-intervention variables that influenced selection likely to be associated with intervention?	NA	NA / Y / PY / PN / N / NI
2.3 **If Y/PY to 2.2**: Were the post-intervention variables that influenced selection likely to be influenced by the outcome or a cause of the outcome?	NA	NA / Y / PY / PN / N / NI
2.4. Do start of follow-up and start of intervention coincide for most participants?	PY. Baseline questionnaire survey were conducted in 2000, 2001, and 2002. The respondents were asked their average weekly hours of LTPA within previous 12 months.	Y / PY / PN / N / NI
2.5. **If Y/PY to 2.2 and 2.3, or N/PN to 2.4**: Were adjustment techniques used that are likely to correct for the presence of selection biases?	NA	NA / Y / PY / PN / N / NI
**Risk of bias judgement**	Low. Data satisfactorily represent the target population and start of follow up and start of intervention coincided for each participant.	Low / Moderate / Serious / Critical / NI
Optional: What is the predicted direction of bias due to selection of participants into the study?		Favours experimental / Favours comparator / Towards null /Away from null / Unpredictable

Bias in classification of interventions		
3.1 Were intervention groups clearly defined?	PY. Strict eligibility criteria for the classification of the exposure (METs), yet PA level was assessed by self-reported questionnaires and the adherence to the intervention is unknown. This is not comparable to a well-performed randomized trial.	Y / PY / PN / N / NI
3.2 Was the information used to define intervention groups recorded at the start of the intervention?	Y. PA level was recorded at the start of the follow up (yet the question describing PA levels refers within the previous 12 months)	Y / PY / PN / N / NI
3.3 Could classification of intervention status have been affected by knowledge of the outcome or risk of the outcome?	N	Y / PY / PN / N / NI
**Risk of bias judgement**	Low. Intervention status is well defined.	Low / Moderate / Serious / Critical / NI
Optional: What is the predicted direction of bias due to classification of interventions?		Favours experimental / Favours comparator / Towards null /Away from null / Unpredictable

Bias due to deviations from intended interventions		
**If your aim for this study is to assess the effect of assignment to intervention, answer questions 4.1 and 4.2**		
4.1. Were there deviations from the intended intervention beyond what would be expected in usual practice?	NA	Y / PY / PN / N / NI
4.2. **If Y/PY to 4.1**: Were these deviations from intended intervention unbalanced between groups *and* likely to have affected the outcome?	NA	NA / Y / PY / PN / N / NI
**If your aim for this study is to assess the effect of starting and adhering to intervention, answer questions 4.3 to 4.6**		
4.3. Were important co-interventions balanced across intervention groups?	PY	Y / PY / PN / N / NI
4.4. Was the intervention implemented successfully for most participants?	PY	Y / PY / PN / N / NI
4.5. Did study participants adhere to the assigned intervention regimen?	NI	Y / PY / PN / N / NI
4.6. **If N/PN to 4.3, 4.4 or 4.5**: Was an appropriate analysis used to estimate the effect of starting and adhering to the intervention?	NA	NA / Y / PY / PN / N / NI
**Risk of bias judgement**	Moderate. the study is sound for a non-randomized study with regard to this domain but cannot be considered comparable to a well-performed randomized trial. It is not clear whether participants adhered to intervention, but their impact on the outcome is expected to be slight.	Low / Moderate / Serious / Critical / NI
Optional: What is the predicted direction of bias due to deviations from the intended interventions?	Unpredictable	Favours experimental / Favours comparator / Towards null /Away from null / Unpredictable

Bias due to missing data		
5.1 Were outcome data available for all, or nearly all, participants?	PN. 26% of participants did not give permission to the mortality linkages.	Y / PY / PN / N / NI
5.2 Were participants excluded due to missing data on intervention status?	Y. Yet, the number of participants reporting no LTPA was low (n=28).	Y / PY / PN / N / NI
5.3 Were participants excluded due to missing data on other variables needed for the analysis?	Y	Y / PY / PN / N / NI
5.4 **If PN/N to 5.1, or Y/PY to 5.2 or 5.3**: Are the proportion of participants and reasons for missing data similar across interventions?	NI. Unknown the proportion of missing data across intervention groups.	NA / Y / PY / PN / N / NI
5.5 **If PN/N to 5.1, or Y/PY to 5.2 or 5.3**: Is there evidence that results were robust to the presence of missing data?	NI	NA / Y / PY / PN / N / NI
**Risk of bias judgement**	Critical. Missing data were not addressed through appropriate analysis.	Low / Moderate / Serious / Critical / NI
Optional: What is the predicted direction of bias due to missing data?	Unpredictable	Favours experimental / Favours comparator / Towards null /Away from null / Unpredictable

Bias in measurement of outcomes		
6.1 Could the outcome measure have been influenced by knowledge of the intervention received?	PN	Y / PY / PN / N / NI
6.2 Were outcome assessors aware of the intervention received by study participants?	PN	Y / PY / PN / N / NI
6.3 Were the methods of outcome assessment comparable across intervention groups?	Y	Y / PY / PN / N / NI
6.4 Were any systematic errors in measurement of the outcome related to intervention received?	PN	Y / PY / PN / N / NI
**Risk of bias judgement**	Low	Low / Moderate / Serious / Critical / NI
Optional: What is the predicted direction of bias due to measurement of outcomes?		Favours experimental / Favours comparator / Towards null /Away from null / Unpredictable

Bias in selection of the reported result		
Is the reported effect estimate likely to be selected, on the basis of the results, from... 7.1. ... multiple outcome *measurements* within the outcome domain?	PN	Y / PY / PN / N / NI
7.2 ... multiple *analyses* of the intervention-outcome relationship?	PN	Y / PY / PN / N / NI
7.3 ... different *subgroups*?	PN	Y / PY / PN / N / NI
**Risk of bias judgement**	Moderate	Low / Moderate / Serious / Critical / NI
Optional: What is the predicted direction of bias due to selection of the reported result?	Unpredictable	Favours experimental / Favours comparator / Towards null /Away from null / Unpredictable

Overall bias		
**Risk of bias judgement**	Critical. The bias judgement for missing data was scored as critical.	Low / Moderate / Serious / Critical / NI
Optional: What is the overall predicted direction of bias for this outcome?	Favour experimental	Favours experimental / Favours comparator / Towards null /Away from null / Unpredictable

This work is licensed under a Creative Commons Attribution-NonCommercial-NoDerivatives 4.0 International License.

Shiroma et al. 2014

Responses underlined in green are potential markers for low risk of bias, and responses in red are potential markers for a risk of bias. Where questions relate only to sign posts to other questions, no formatting is used.

Signalling questions	Description	Response options
**Bias due to confounding**		
1.1 Is there potential for confounding of the effect of intervention in this study?<br>**If N/PN to 1.1:** the study can be considered to be at low risk of bias due to confounding and no further signalling questions need be considered	PY	Y / PY / PN / N
**If Y/PY to 1.1**: determine whether there is a need to assess time-varying confounding:	N	
1.2. Was the analysis based on splitting participants' follow up time according to intervention received?<br>**If N/PN**, answer questions relating to baseline confounding (1.4 to 1.6)<br>**If Y/PY**, go to question 1.3.	NA	NA / Y / PY / PN / N / NI
1.3. Were intervention discontinuations or switches likely to be related to factors that are prognostic for the outcome?<br>**If N/PN**, answer questions relating to baseline confounding (1.4 to 1.6)<br>**If Y/PY**, answer questions relating to both baseline and time-varying confounding (1.7 and 1.8)	NA	NA / Y / PY / PN / N / NI

**Questions relating to baseline confounding only**		
1.4. Did the authors use an appropriate analysis method that controlled for all the important confounding domains?	PN. Insufficient adjustment in the main reported analyses. Other chronic diseases and illness (beside cancer, cvd and diabetes) were not included as confounders.	NA / Y / PY / PN / N / NI
1.5. **If Y/PY to 1.4**: Were confounding domains that were controlled for measured validly and reliably by the variables available in this study?	PN. Confounders were subjectively measured by participants. Residual confounding is likely	NA / Y / PY / PN / N / NI
1.6. Did the authors control for any post-intervention variables that could have been affected by the intervention?	N	NA / Y / PY / PN / N / NI
**Questions relating to baseline and time-varying confounding**		
1.7. Did the authors use an appropriate analysis method that controlled for all the important confounding domains and for time-varying confounding?	NA	NA / Y / PY / PN / N / NI
1.8. **If Y/PY to 1.7**: Were confounding domains that were controlled for measured validly and reliably by the variables available in this study?	NA	NA / Y / PY / PN / N / NI
**Risk of bias judgement**	Moderate	Low / Moderate / Serious / Critical / NI
Optional: What is the predicted direction of bias due to confounding?		Favours experimental / Favours comparator / Unpredictable

Bias in selection of participants into the study		
2.1. Was selection of participants into the study (or into the analysis) based on participant characteristics observed after the start of intervention?<br>**If N/PN to 2.1:** go to 2.4	N	Y / PY / PN / N / NI
2.2. **If Y/PY to 2.1**: Were the post-intervention variables that influenced selection likely to be associated with intervention?	NA	NA / Y / PY / PN / N / NI
2.3 **If Y/PY to 2.2**: Were the post-intervention variables that influenced selection likely to be influenced by the outcome or a cause of the outcome?	NA	NA / Y / PY / PN / N / NI
2.4. Do start of follow-up and start of intervention coincide for most participants?	PY.	Y / PY / PN / N / NI
2.5. **If Y/PY to 2.2 and 2.3, or N/PN to 2.4**: Were adjustment techniques used that are likely to correct for the presence of selection biases?	NA	NA / Y / PY / PN / N / NI
**Risk of bias judgement**	Low.	Low / Moderate / Serious / Critical / NI
Optional: What is the predicted direction of bias due to selection of participants into the study?		Favours experimental / Favours comparator / Towards null /Away from null / Unpredictable

Bias in classification of interventions		
3.1 Were intervention groups clearly defined?	PY. Strict eligibility criteria for the classification of the exposure (METs), yet PA level was assessed by self-reported questionnaires and the adherence to the intervention is unknown. The, it is not comparable to a well-performed randomized trial.	Y / PY / PN / N / NI
3.2 Was the information used to define intervention groups recorded at the start of the intervention?	Y. PA level was recorded at the start of the follow up.	Y / PY / PN / N / NI
3.3 Could classification of intervention status have been affected by knowledge of the outcome or risk of the outcome?	N	Y / PY / PN / N / NI
**Risk of bias judgement**	Low	Low / Moderate / Serious / Critical / NI
Optional: What is the predicted direction of bias due to classification of interventions?		Favours experimental / Favours comparator / Towards null /Away from null / Unpredictable

Bias due to deviations from intended interventions		
**If your aim for this study is to assess the effect of assignment to intervention, answer questions 4.1 and 4.2**		
4.1. Were there deviations from the intended intervention beyond what would be expected in usual practice?	NA	Y / PY / PN / N / NI
4.2. **If Y/PY to 4.1**: Were these deviations from intended intervention unbalanced between groups *and* likely to have affected the outcome?	NA	NA / Y / PY / PN / N / NI
**If your aim for this study is to assess the effect of starting and adhering to intervention, answer questions 4.3 to 4.6**		
4.3. Were important co-interventions balanced across intervention groups?	PY	Y / PY / PN / N / NI
4.4. Was the intervention implemented successfully for most participants?	PY	Y / PY / PN / N / NI
4.5. Did study participants adhere to the assigned intervention regimen?	NI. Although PA was collected for the baseline (1988) and 2 subsequent waves (1993 and 1998), it was not clear which measure of PA they used (cumulative average PA or baseline only?). Therefore, its not clear whether participants adhere to PA over time.	Y / PY / PN / N / NI
4.6. **If N/PN to 4.3, 4.4 or 4.5**: Was an appropriate analysis used to estimate the effect of starting and adhering to the intervention?	NA	NA / Y / PY / PN / N / NI
**Risk of bias judgement**	Moderate. the study is sound for a non-randomized study with regard to this domain but cannot be considered comparable to a well-performed randomized trial. It is not clear whether participants adhered to intervention, but their impact on the outcome is expected to be slight.	Low / Moderate / Serious / Critical / NI
Optional: What is the predicted direction of bias due to deviations from the intended interventions?	Unpredictable	Favours experimental / Favours comparator / Towards null /Away from null / Unpredictable

Bias due to missing data		
5.1 Were outcome data available for all, or nearly all, participants?	NI. Authors did not provide information about this question.	Y / PY / PN / N / NI
5.2 Were participants excluded due to missing data on intervention status?	Y	Y / PY / PN / N / NI
5.3 Were participants excluded due to missing data on other variables needed for the analysis?	PN	Y / PY / PN / N / NI
5.4 **If PN/N to 5.1, or Y/PY to 5.2 or 5.3**: Are the proportion of participants and reasons for missing data similar across interventions?	NI. Unknown the proportion of missing data across intervention groups.	NA / Y / PY / PN / N / NI
5.5 **If PN/N to 5.1, or Y/PY to 5.2 or 5.3**: Is there evidence that results were robust to the presence of missing data?	NI	NA / Y / PY / PN / N / NI
**Risk of bias judgement**	NI	Low / Moderate / Serious / Critical / NI
Optional: What is the predicted direction of bias due to missing data?	Unpredictable	Favours experimental / Favours comparator / Towards null /Away from null / Unpredictable

Bias in measurement of outcomes		
6.1 Could the outcome measure have been influenced by knowledge of the intervention received?	PN	Y / PY / PN / N / NI
6.2 Were outcome assessors aware of the intervention received by study participants?	PN	Y / PY / PN / N / NI
6.3 Were the methods of outcome assessment comparable across intervention groups?	Y	Y / PY / PN / N / NI
6.4 Were any systematic errors in measurement of the outcome related to intervention received?	PN	Y / PY / PN / N / NI
**Risk of bias judgement**	Low	Low / Moderate / Serious / Critical / NI
Optional: What is the predicted direction of bias due to measurement of outcomes?		Favours experimental / Favours comparator / Towards null /Away from null / Unpredictable

Bias in selection of the reported result		
Is the reported effect estimate likely to be selected, on the basis of the results, from...		
7.1. ... multiple outcome *measurements* within the outcome domain?	N	Y / PY / PN / N / NI
7.2 ... multiple *analyses* of the intervention-outcome relationship?	PN.	Y / PY / PN / N / NI
7.3 ... different *subgroups*?	PN. Detailed subgroups analyses are not shown.	Y / PY / PN / N / NI
**Risk of bias judgement**	Low	Low / Moderate / Serious / Critical / NI
Optional: What is the predicted direction of bias due to selection of the reported result?	Unpredictable	Favours experimental / Favours comparator / Towards null /Away from null / Unpredictable

Overall bias		
**Risk of bias judgement**	Moderate	Low / Moderate / Serious / Critical / NI
Optional: What is the overall predicted direction of bias for this outcome?	Unpredictable	Favours experimental / Favours comparator / Towards null /Away from null / Unpredictable

This work is licensed under a Creative Commons Attribution-NonCommercial-NoDerivatives 4.0 International License.

Gebel et al. 2015

Responses underlined in green are potential markers for low risk of bias, and responses in red are potential markers for a risk of bias. Where questions relate only to sign posts to other questions, no formatting is used.

Signalling questions	Description	Response options
**Bias due to confounding**		
1.1 Is there potential for confounding of the effect of intervention in this study?<br>**If N/PN to 1.1:** the study can be considered to be at low risk of bias due to confounding and no further signalling questions need be considered	Y. CMD are not adjusted in main results (all, by sex), weight status (not obese include overweight).	Y / PY / PN / N
**If Y/PY to 1.1**: determine whether there is a need to assess time-varying confounding:		
1.2. Was the analysis based on splitting participants' follow up time according to intervention received?<br>**If N/PN**, answer questions relating to baseline confounding (1.4 to 1.6)<br>**If Y/PY**, go to question 1.3.	N	NA / Y / PY / PN / N / NI
1.3. Were intervention discontinuations or switches likely to be related to factors that are prognostic for the outcome?<br>**If N/PN**, answer questions relating to baseline confounding (1.4 to 1.6)<br>**If Y/PY**, answer questions relating to both baseline and time-varying confounding (1.7 and 1.8)	NA	NA / Y / PY / PN / N / NI

**Questions relating to baseline confounding only**		
1.4. Did the authors use an appropriate analysis method that controlled for all the important confounding domains?	N. Insufficient adjustment in the main reported analyses. Cardiometabolic diseases, BMI (three categories), other illnesses were not included as confounders in the main reported findings.	NA / Y / PY / PN / N / NI
1.5. **If Y/PY to 1.4**: Were confounding domains that were controlled for measured validly and reliably by the variables available in this study?	NA	NA / Y / PY / PN / N / NI
1.6. Did the authors control for any post-intervention variables that could have been affected by the intervention?	N	NA / Y / PY / PN / N / NI
**Questions relating to baseline and time-varying confounding**		
1.7. Did the authors use an appropriate analysis method that controlled for all the important confounding domains and for time-varying confounding?	NA	NA / Y / PY / PN / N / NI
1.8. **If Y/PY to 1.7**: Were confounding domains that were controlled for measured validly and reliably by the variables available in this study?	NA	NA / Y / PY / PN / N / NI
**Risk of bias judgement**	Serious.	Low / Moderate / Serious / Critical / NI
Optional: What is the predicted direction of bias due to confounding?	Favours experimental	Favours experimental / Favours comparator / Unpredictable

Bias in selection of participants into the study		
2.1. Was selection of participants into the study (or into the analysis) based on participant characteristics observed after the start of intervention?<br>**If N/PN to 2.1:** go to 2.4	N	Y / PY / PN / N / NI
2.2. **If Y/PY to 2.1**: Were the post-intervention variables that influenced selection likely to be associated with intervention?	NA	NA / Y / PY / PN / N / NI
2.3 **If Y/PY to 2.2**: Were the post-intervention variables that influenced selection likely to be influenced by the outcome or a cause of the outcome?	NA	NA / Y / PY / PN / N / NI
2.4. Do start of follow-up and start of intervention coincide for most participants?	Y	Y / PY / PN / N / NI
2.5. **If Y/PY to 2.2 and 2.3, or N/PN to 2.4**: Were adjustment techniques used that are likely to correct for the presence of selection biases?	NA	NA / Y / PY / PN / N / NI
**Risk of bias judgement**	Low	Low / Moderate / Serious / Critical / NI
Optional: What is the predicted direction of bias due to selection of participants into the study?		Favours experimental / Favours comparator / Towards null /Away from null / Unpredictable

Bias in classification of interventions		
3.1 Were intervention groups clearly defined?	PY. Strict eligibility criteria for the classification of the exposure (METs), yet PA level was assessed by self-reported questionnaires and the adherence to the intervention is unknown. This is not comparable to a well-performed randomized trial.	Y / PY / PN / N / NI
3.2 Was the information used to define intervention groups recorded at the start of the intervention?	Y. PA level was recorded at the start of the follow up (last week before questionnaires were completed)	Y / PY / PN / N / NI
3.3 Could classification of intervention status have been affected by knowledge of the outcome or risk of the outcome?	PY – mean follow-up of 6.5 years and did not adjusted for chronic diseases and illness at the baseline – reverse causation is likely	Y / PY / PN / N / NI
**Risk of bias judgement**	Low	Low / Moderate / Serious / Critical / NI
Optional: What is the predicted direction of bias due to classification of interventions?		Favours experimental / Favours comparator / Towards null /Away from null / Unpredictable

Bias due to deviations from intended interventions		
**If your aim for this study is to assess the effect of assignment to intervention, answer questions 4.1 and 4.2**		
4.1. Were there deviations from the intended intervention beyond what would be expected in usual practice?	NA	Y / PY / PN / N / NI
4.2. **If Y/PY to 4.1**: Were these deviations from intended intervention unbalanced between groups *and* likely to have affected the outcome?	NA	NA / Y / PY / PN / N / NI
**If your aim for this study is to assess the effect of starting and adhering to intervention, answer questions 4.3 to 4.6**		
4.3. Were important co-interventions balanced across intervention groups?	PY	Y / PY / PN / N / NI
4.4. Was the intervention implemented successfully for most participants?	PY	Y / PY / PN / N / NI
4.5. Did study participants adhere to the assigned intervention regimen?	NI	Y / PY / PN / N / NI
4.6. **If N/PN to 4.3, 4.4 or 4.5**: Was an appropriate analysis used to estimate the effect of starting and adhering to the intervention?	NA	NA / Y / PY / PN / N / NI
**Risk of bias judgement**	Moderate	Low / Moderate / Serious / Critical / NI
Optional: What is the predicted direction of bias due to deviations from the intended interventions?	Unpredictable	Favours experimental / Favours comparator / Towards null /Away from null / Unpredictable

Bias due to missing data		
5.1 Were outcome data available for all, or nearly all, participants?	NI. Authors did not clarify this question.	Y / PY / PN / N / NI
5.2 Were participants excluded due to missing data on intervention status?	Y	Y / PY / PN / N / NI
5.3 Were participants excluded due to missing data on other variables needed for the analysis?	N	Y / PY / PN / N / NI
5.4 **If PN/N to 5.1, or Y/PY to 5.2 or 5.3**: Are the proportion of participants and reasons for missing data similar across interventions?	NI. Unknown the proportion of missing data across intervention groups.	NA / Y / PY / PN / N / NI
5.5 **If PN/N to 5.1, or Y/PY to 5.2 or 5.3**: Is there evidence that results were robust to the presence of missing data?	NI	NA / Y / PY / PN / N / NI
**Risk of bias judgement**	NI - No information is reported about missing data or the potential for data to be missing.	Low / Moderate / Serious / Critical / NI
Optional: What is the predicted direction of bias due to missing data?	Unpredictable	Favours experimental / Favours comparator / Towards null /Away from null / Unpredictable

Bias in measurement of outcomes		
6.1 Could the outcome measure have been influenced by knowledge of the intervention received?	PN	Y / PY / PN / N / NI
6.2 Were outcome assessors aware of the intervention received by study participants?	NI	Y / PY / PN / N / NI
6.3 Were the methods of outcome assessment comparable across intervention groups?	Y	Y / PY / PN / N / NI
6.4 Were any systematic errors in measurement of the outcome related to intervention received?	PN	Y / PY / PN / N / NI
**Risk of bias judgement**	Low	Low / Moderate / Serious / Critical / NI
Optional: What is the predicted direction of bias due to measurement of outcomes?		Favours experimental / Favours comparator / Towards null /Away from null / Unpredictable

Bias in selection of the reported result		
Is the reported effect estimate likely to be selected, on the basis of the results, from...		
7.1. ... multiple outcome *measurements* within the outcome domain?	N	Y / PY / PN / N / NI
7.2 ... multiple *analyses* of the intervention-outcome relationship?	PN	Y / PY / PN / N / NI
7.3 ... different *subgroups*?	PY. Main findings were based on models that excluded cardiometabolic diseases and BMI as confounders, despite they included it in the stratified analyses	Y / PY / PN / N / NI
**Risk of bias judgement**	Moderate the reported results excluding CMD and BMI did not appear to be reported on the basis of the results (see stratified analyses)	Low / Moderate / Serious / Critical / NI
Optional: What is the predicted direction of bias due to selection of the reported result?	Favour experimental.	Favours experimental / Favours comparator / Towards null /Away from null / Unpredictable

Overall bias		
**Risk of bias judgement**	Serious.	Low / Moderate / Serious / Critical / NI
Optional: What is the overall predicted direction of bias for this outcome?	Favour experimental	Favours experimental / Favours comparator / Towards null /Away from null / Unpredictable

This work is licensed under a Creative Commons Attribution-NonCommercial-NoDerivatives 4.0 International License.

Kikuchi et al. 2018

Responses underlined in green are potential markers for low risk of bias, and responses in red are potential markers for a risk of bias. Where questions relate only to sign posts to other questions, no formatting is used.

Signalling questions	Description	Response options
**Bias due to confounding**		
1.1 Is there potential for confounding of the effect of intervention in this study?<br>**If N/PN to 1.1:** the study can be considered to be at low risk of bias due to confounding and no further signalling questions need be considered	Y. Social class or dietary factors were not included as covariates.	Y / PY / PN / N
**If Y/PY to 1.1**: determine whether there is a need to assess time-varying confounding:		
1.2. Was the analysis based on splitting participants' follow up time according to intervention received?<br>**If N/PN**, answer questions relating to baseline confounding (1.4 to 1.6)<br>**If Y/PY**, go to question 1.3.	N	NA / Y / PY / PN / N / NI
1.3. Were intervention discontinuations or switches likely to be related to factors that are prognostic for the outcome?<br>**If N/PN**, answer questions relating to baseline confounding (1.4 to 1.6)<br>**If Y/PY**, answer questions relating to both baseline and time-varying confounding (1.7 and 1.8)	NA	NA / Y / PY / PN / N / NI

**Questions relating to baseline confounding only**		
1.4. Did the authors use an appropriate analysis method that controlled for all the important confounding domains?	N. Insufficient adjustment in the main reported analyses. Dietary factors or socioeconomic status were not included as confounders in the main reported findings.	NA / Y / PY / PN / N / NI
1.5. **If Y/PY to 1.4**: Were confounding domains that were controlled for measured validly and reliably by the variables available in this study?	NA	NA / Y / PY / PN / N / NI
1.6. Did the authors control for any post-intervention variables that could have been affected by the intervention?	N	NA / Y / PY / PN / N / NI
**Questions relating to baseline and time-varying confounding**		
1.7. Did the authors use an appropriate analysis method that controlled for all the important confounding domains and for time-varying confounding?	NA	NA / Y / PY / PN / N / NI
1.8. **If Y/PY to 1.7**: Were confounding domains that were controlled for measured validly and reliably by the variables available in this study?	NA	NA / Y / PY / PN / N / NI
**Risk of bias judgement**	Serious.	Low / Moderate / Serious / Critical / NI
Optional: What is the predicted direction of bias due to confounding?	Favours experimental	Favours experimental / Favours comparator / Unpredictable

Bias in selection of participants into the study		
2.1. Was selection of participants into the study (or into the analysis) based on participant characteristics observed after the start of intervention?<br>**If N/PN to 2.1:** go to 2.4	N	Y / PY / PN / N / NI
2.2. **If Y/PY to 2.1**: Were the post-intervention variables that influenced selection likely to be associated with intervention?	NA	NA / Y / PY / PN / N / NI
2.3 **If Y/PY to 2.2**: Were the post-intervention variables that influenced selection likely to be influenced by the outcome or a cause of the outcome?	NA	NA / Y / PY / PN / N / NI
2.4. Do start of follow-up and start of intervention coincide for most participants?	Y	Y / PY / PN / N / NI
2.5. **If Y/PY to 2.2 and 2.3, or N/PN to 2.4**: Were adjustment techniques used that are likely to correct for the presence of selection biases?	NA	NA / Y / PY / PN / N / NI
**Risk of bias judgement**	Low	Low / Moderate / Serious / Critical / NI
Optional: What is the predicted direction of bias due to selection of participants into the study?		Favours experimental / Favours comparator / Towards null /Away from null / Unpredictable

Bias in classification of interventions		
3.1 Were intervention groups clearly defined?	PY. Strict eligibility criteria for the classification of the exposure (METs), yet PA level was assessed by self-reported questionnaires and the adherence to the intervention is unknown. This is not comparable to a well-performed randomized trial.	Y / PY / PN / N / NI
3.2 Was the information used to define intervention groups recorded at the start of the intervention?	Y. PA level was recorded at the start of the follow up.	Y / PY / PN / N / NI
3.3 Could classification of intervention status have been affected by knowledge of the outcome or risk of the outcome?	PN	Y / PY / PN / N / NI
**Risk of bias judgement**	Low	Low / Moderate / Serious / Critical / NI
Optional: What is the predicted direction of bias due to classification of interventions?		Favours experimental / Favours comparator / Towards null /Away from null / Unpredictable

Bias due to deviations from intended interventions		
**If your aim for this study is to assess the effect of assignment to intervention, answer questions 4.1 and 4.2**		
4.1. Were there deviations from the intended intervention beyond what would be expected in usual practice?	NA	Y / PY / PN / N / NI
4.2. **If Y/PY to 4.1**: Were these deviations from intended intervention unbalanced between groups *and* likely to have affected the outcome?	NA	NA / Y / PY / PN / N / NI
**If your aim for this study is to assess the effect of starting and adhering to intervention, answer questions 4.3 to 4.6**		
4.3. Were important co-interventions balanced across intervention groups?	PY	Y / PY / PN / N / NI
4.4. Was the intervention implemented successfully for most participants?	PY	Y / PY / PN / N / NI
4.5. Did study participants adhere to the assigned intervention regimen?	NI	Y / PY / PN / N / NI
4.6. **If N/PN to 4.3, 4.4 or 4.5**: Was an appropriate analysis used to estimate the effect of starting and adhering to the intervention?	NA	NA / Y / PY / PN / N / NI
**Risk of bias judgement**	Moderate	Low / Moderate / Serious / Critical / NI
Optional: What is the predicted direction of bias due to deviations from the intended interventions?	Unpredictable	Favours experimental / Favours comparator / Towards null /Away from null / Unpredictable

Bias due to missing data		
5.1 Were outcome data available for all, or nearly all, participants?	NI	Y / PY / PN / N / NI
5.2 Were participants excluded due to missing data on intervention status?	NI	Y / PY / PN / N / NI
5.3 Were participants excluded due to missing data on other variables needed for the analysis?	Y	Y / PY / PN / N / NI
5.4 **If PN/N to 5.1, or Y/PY to 5.2 or 5.3**: Are the proportion of participants and reasons for missing data similar across interventions?	NI. Unknown the proportion of missing data across intervention groups.	NA / Y / PY / PN / N / NI
5.5 **If PN/N to 5.1, or Y/PY to 5.2 or 5.3**: Is there evidence that results were robust to the presence of missing data?	NI. The only information reported was: "By using data from the second survey, we observed no significant differences in baseline characteristics between responders and nonresponders in the third survey." But this does not provide evidence that results (associations) were robust to the presence of missing data.	NA / Y / PY / PN / N / NI
**Risk of bias judgement**	NI	Low / Moderate / Serious / Critical / NI
Optional: What is the predicted direction of bias due to missing data?	Unpredictable	Favours experimental / Favours comparator / Towards null /Away from null / Unpredictable

Bias in measurement of outcomes		
6.1 Could the outcome measure have been influenced by knowledge of the intervention received?	PN	Y / PY / PN / N / NI
6.2 Were outcome assessors aware of the intervention received by study participants?	PN	Y / PY / PN / N / NI
6.3 Were the methods of outcome assessment comparable across intervention groups?	Y	Y / PY / PN / N / NI
6.4 Were any systematic errors in measurement of the outcome related to intervention received?	PN	Y / PY / PN / N / NI
**Risk of bias judgement**	Low	Low / Moderate / Serious / Critical / NI
Optional: What is the predicted direction of bias due to measurement of outcomes?		Favours experimental / Favours comparator / Towards null /Away from null / Unpredictable

Bias in selection of the reported result		
Is the reported effect estimate likely to be selected, on the basis of the results, from...		
7.1. ... multiple outcome *measurements* within the outcome domain?	PN	Y / PY / PN / N / NI
7.2 ... multiple *analyses* of the intervention-outcome relationship?	PN	Y / PY / PN / N / NI
7.3 ... different *subgroups*?	PN. Main findings were based on the whole group analyses, but authors also reported analyses by sex and age, which showed less evidence of mortality benefits in elderly women. These analyses were not a priori plan.	Y / PY / PN / N / NI
**Risk of bias judgement**	Low	Low / Moderate / Serious / Critical / NI
Optional: What is the predicted direction of bias due to selection of the reported result?		Favours experimental / Favours comparator / Towards null /Away from null / Unpredictable

**Overall bias**		
**Risk of bias judgement**	Serious	Low / Moderate / Serious / Critical / NI
Optional: What is the overall predicted direction of bias for this outcome?	Favour experimental	Favours experimental / Favours comparator / Towards null /Away from null / Unpredictable

This work is licensed under a Creative Commons Attribution-NonCommercial-NoDerivatives 4.0 International License.

Rey-Lopez et al. 2019

Responses underlined in green are potential markers for low risk of bias, and responses in red are potential markers for a risk of bias. Where questions relate only to sign posts to other questions, no formatting is used.

Signalling questions	Description	Response options
**Bias due to confounding**		
1.1 Is there potential for confounding of the effect of intervention in this study?<br>**If N/PN to 1.1:** the study can be considered to be at low risk of bias due to confounding and no further signalling questions need be considered	PY. dietary factors were not included as covariates.	Y / PY / PN / N
**If Y/PY to 1.1**: determine whether there is a need to assess time-varying confounding:		
1.2. Was the analysis based on splitting participants' follow up time according to intervention received?<br>**If N/PN**, answer questions relating to baseline confounding (1.4 to 1.6)<br>**If Y/PY**, go to question 1.3.	N	NA / Y / PY / PN / N / NI
1.3. Were intervention discontinuations or switches likely to be related to factors that are prognostic for the outcome?<br>**If N/PN**, answer questions relating to baseline confounding (1.4 to 1.6)<br>**If Y/PY**, answer questions relating to both baseline and time-varying confounding (1.7 and 1.8)	NA	NA / Y / PY / PN / N / NI

**Questions relating to baseline confounding only**		
1.4. Did the authors use an appropriate analysis method that controlled for all the important confounding domains?	N. Insufficient adjustment in the main reported analyses. Dietary factors were not included as confounders in the main reported findings.	NA / Y / PY / PN / N / NI
1.5. **If Y/PY to 1.4**: Were confounding domains that were controlled for measured validly and reliably by the variables available in this study?	NA	NA / Y / PY / PN / N / NI
1.6. Did the authors control for any post-intervention variables that could have been affected by the intervention?	N	NA / Y / PY / PN / N / NI
**Questions relating to baseline and time-varying confounding**		
1.7. Did the authors use an appropriate analysis method that controlled for all the important confounding domains and for time-varying confounding?	NA	NA / Y / PY / PN / N / NI
1.8. **If Y/PY to 1.7**: Were confounding domains that were controlled for measured validly and reliably by the variables available in this study?	NA	NA / Y / PY / PN / N / NI
**Risk of bias judgement**	Serious.	Low / Moderate / Serious / Critical / NI
Optional: What is the predicted direction of bias due to confounding?	Favours experimental	Favours experimental / Favours comparator / Unpredictable

Bias in selection of participants into the study		
2.1. Was selection of participants into the study (or into the analysis) based on participant characteristics observed after the start of intervention?<br>**If N/PN to 2.1:** go to 2.4	N	Y / PY / PN / N / NI
2.2. **If Y/PY to 2.1**: Were the post-intervention variables that influenced selection likely to be associated with intervention?	NA	NA / Y / PY / PN / N / NI
2.3 **If Y/PY to 2.2**: Were the post-intervention variables that influenced selection likely to be influenced by the outcome or a cause of the outcome?	NA	NA / Y / PY / PN / N / NI
2.4. Do start of follow-up and start of intervention coincide for most participants?	Y	Y / PY / PN / N / NI
2.5. **If Y/PY to 2.2 and 2.3, or N/PN to 2.4**: Were adjustment techniques used that are likely to correct for the presence of selection biases?	NA	NA / Y / PY / PN / N / NI
**Risk of bias judgement**	Low	Low / Moderate / Serious / Critical / NI
Optional: What is the predicted direction of bias due to selection of participants into the study?		Favours experimental / Favours comparator / Towards null /Away from null / Unpredictable

Bias in classification of interventions		
3.1 Were intervention groups clearly defined?	PY. Strict eligibility criteria for the classification of the exposure (METs), yet PA level was assessed by self-reported questionnaires and the adherence to the intervention is unknown. This is not comparable to a well-performed randomized trial.	Y / PY / PN / N / NI
3.2 Was the information used to define intervention groups recorded at the start of the intervention?	Y. PA level was recorded at the start of the follow up.	Y / PY / PN / N / NI
3.3 Could classification of intervention status have been affected by knowledge of the outcome or risk of the outcome?	PN	Y / PY / PN / N / NI
**Risk of bias judgement**	Low	Low / Moderate / Serious / Critical / NI
Optional: What is the predicted direction of bias due to classification of interventions?		Favours experimental / Favours comparator / Towards null /Away from null / Unpredictable

Bias due to deviations from intended interventions		
**If your aim for this study is to assess the effect of assignment to intervention, answer questions 4.1 and 4.2**		
4.1. Were there deviations from the intended intervention beyond what would be expected in usual practice?	NA	Y / PY / PN / N / NI
4.2. **If Y/PY to 4.1**: Were these deviations from intended intervention unbalanced between groups *and* likely to have affected the outcome?	NA	NA / Y / PY / PN / N / NI
**If your aim for this study is to assess the effect of starting and adhering to intervention, answer questions 4.3 to 4.6**		
4.3. Were important co-interventions balanced across intervention groups?	PY	Y / PY / PN / N / NI
4.4. Was the intervention implemented successfully for most participants?	PY	Y / PY / PN / N / NI
4.5. Did study participants adhere to the assigned intervention regimen?	NI	Y / PY / PN / N / NI
4.6. **If N/PN to 4.3, 4.4 or 4.5**: Was an appropriate analysis used to estimate the effect of starting and adhering to the intervention?	NA	NA / Y / PY / PN / N / NI
**Risk of bias judgement**	Moderate	Low / Moderate / Serious / Critical / NI
Optional: What is the predicted direction of bias due to deviations from the intended interventions?	Unpredictable	Favours experimental / Favours comparator / Towards null /Away from null / Unpredictable

Bias due to missing data		
5.1 Were outcome data available for all, or nearly all, participants?	NI	Y / PY / PN / N / NI
5.2 Were participants excluded due to missing data on intervention status?	NI	Y / PY / PN / N / NI
5.3 Were participants excluded due to missing data on other variables needed for the analysis?	Y	Y / PY / PN / N / NI
5.4 **If PN/N to 5.1, or Y/PY to 5.2 or 5.3**: Are the proportion of participants and reasons for missing data similar across interventions?	NI. Unknown the proportion of missing data across intervention groups.	NA / Y / PY / PN / N / NI
5.5 **If PN/N to 5.1, or Y/PY to 5.2 or 5.3**: Is there evidence that results were robust to the presence of missing data?	NI.	NA / Y / PY / PN / N / NI
**Risk of bias judgement**	NI	Low / Moderate / Serious / Critical / NI
Optional: What is the predicted direction of bias due to missing data?	Unpredictable	Favours experimental / Favours comparator / Towards null /Away from null / Unpredictable

Bias in measurement of outcomes		
6.1 Could the outcome measure have been influenced by knowledge of the intervention received?	PN	Y / PY / PN / N / NI
6.2 Were outcome assessors aware of the intervention received by study participants?	PN	Y / PY / PN / N / NI
6.3 Were the methods of outcome assessment comparable across intervention groups?	Y	Y / PY / PN / N / NI
6.4 Were any systematic errors in measurement of the outcome related to intervention received?	PN	Y / PY / PN / N / NI
**Risk of bias judgement**	Low	Low / Moderate / Serious / Critical / NI
Optional: What is the predicted direction of bias due to measurement of outcomes?		Favours experimental / Favours comparator / Towards null /Away from null / Unpredictable

Bias in selection of the reported result		
Is the reported effect estimate likely to be selected, on the basis of the results, from...		
7.1. ... multiple outcome *measurements* within the outcome domain?	PN	Y / PY / PN / N / NI
7.2 ... multiple *analyses* of the intervention-outcome relationship?	PN	Y / PY / PN / N / NI
7.3 ... different *subgroups*?	PN. Authors reported multiple analyses: i.e. results by sex, age, BMI, physical activity, employing different multivariate models; and using sensitivity analyses (excluding deaths during the first 24 months of follow-up) and the direction of the findings did not change.	Y / PY / PN / N / NI
**Risk of bias judgement**	Low	Low / Moderate / Serious / Critical / NI
Optional: What is the predicted direction of bias due to selection of the reported result?		Favours experimental / Favours comparator / Towards null /Away from null / Unpredictable

Overall bias		
**Risk of bias judgement**	Serious	Low / Moderate / Serious / Critical / NI
Optional: What is the overall predicted direction of bias for this outcome?	Favour experimental	Favours experimental / Favours comparator / Towards null /Away from null / Unpredictable

This work is licensed under a Creative Commons Attribution-NonCommercial-NoDerivatives 4.0 International License.
