# Supplementary material for: Do vigorous-intensity and moderate-intensity physical activities reduce mortality to the same extent? A systematic review and meta-analysis
Source: BMJ Open Sport Exerc Med. 2020 Oct 5;6(1):e000775. doi: 10.1136/bmjsem-2020-000775 (PMC7610342; doi:10.1136/bmjsem-2020-000775)

Appendix D: Forest plot summary of association between vigorous and moderate intensity physical activity and all-cause mortality (after the exclusion of Lahti et al. 2014)

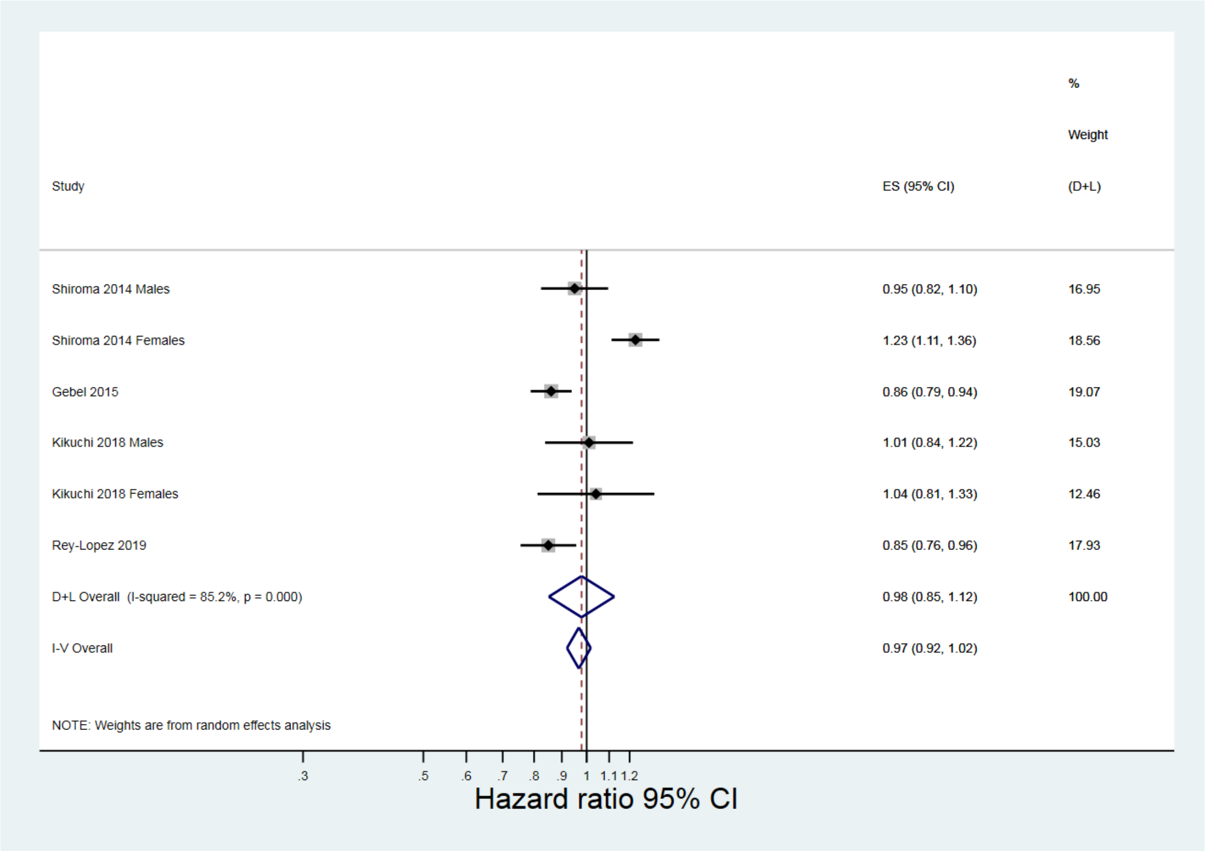

Supplement: Supplementary data [file bmjsem-2020-000775supp004.pdf]
